# Enhanced Activity of Leather Materials Coated with Silver, Copper and Graphene Oxides-Decorated TiO_2_ Nanocomposites and Gamma Irradiated

**DOI:** 10.3390/ma19112358

**Published:** 2026-06-02

**Authors:** Carmen Gaidau, Cosmin Alexe, Rodica Roxana Constantinescu, Laurentiu Dinca, Ioana Stanculescu, Mihalis Cutrubinis, Dragoș Cosma

**Affiliations:** 1 The Research and Development National Institute for Textiles and Leather, 16, Lucretiu Patrascanu Street, 030508 Bucharest, Romania; cosminandrei.alexe@yahoo.com (C.A.); rodica.roxana@yahoo.com (R.R.C.); laurentiu.dinca@incdtp.ro (L.D.); 2 Department of Physical Chemistry, University of Bucharest, 4–12 Regina Elisabeta Bd., 030018 Bucharest, Romania; 3 Horia Hulubei National Institute of Research and Development for Physics and Nuclear Engineering, 30 Reactorului Str., 077125 Magurele, Romania; mcutrubinis@nipne.ro; 4 National Institute for Research and Development of Isotopic and Molecular Technologies, 67–103 Donat Street, 400335 Cluj-Napoca, Romania; dragos.cosma@itim-cj.ro

**Keywords:** antibacterial surfaces, leather surface, composite nanoparticles, titanium dioxide decorated with Ag, Cu_2_O/CuO, GO, gamma irradiation

## Abstract

It is known that antimicrobial surface treatments are one of the measures that can reduce the spread of viral, bacterial or fungal infections that threaten humans’ life. The excessive use of antibiotics has led to the emergence of resistant microorganisms. This study presents the potential of nanocomposites to impart durable antimicrobial properties, which can be enhanced by the use of gamma radiation, even after two months of activation. Leather surfaces finished with composite titanium dioxide nanoparticles decorated with silver, copper oxide and/or graphene oxide were investigated before, immediately after, and after 60 days of gamma irradiation treatment. The antibacterial activity against *Escherichia coli* ATCC 25922 and *Staphylococcus aureus* ATCC 6538 was found to be maintained, and even slightly increased over time compared to unirradiated leathers. Analyses of the morphology and composition of the surface of treated leathers using SEM/EDS, ATR/FTIR, as well as photo activity tests allowed the identification of structural characteristics and the modifications induced by gamma radiation activation. Evaluating the resistance properties of leathers finished with the new nanoparticle composites compared to those finished classically confirmed the quality of the applied technologies. These results provide a solution for antimicrobial treatment of medical equipment, including footwear, with potential applications in other areas such as furniture, aircraft or car upholstery, clothing and bags.

## 1. Introduction

Leather is extensively used in a variety of applications, ranging from everyday garments, bags and furniture upholstery to medical footwear. However, it remains vulnerable to microbial contamination and organic fouling [1,2]. Enhancing the functionality of leather with nanostructured coatings is an effective strategy for tackling these challenges. Titanium dioxide nanoparticles (TiO_2_ NPs) and TiO_2_ nanotubes (TNTs) are promising due to their photocatalytic ability to generate reactive oxygen species (ROS), which are effective against a wide range of microbes and pollutants [3,4,5]. However, the limited activity of pristine TiO_2_ NPs under visible light has driven the development of composite coatings that incorporate noble and transition metals. Silver (Ag) and copper (Cu) nanoparticles are well known for their potent antimicrobial action, which involves disrupting membranes and inducing oxidative stress. They are frequently combined with TiO_2_ NPs to achieve enhanced effects [6,7]. Meanwhile, graphene oxide (GO) plays a dual role in improving nanoparticles dispersion and stability [1]. However, some studies suggest that GO may hinder TiO_2_ activity, depending on the synthesis method employed [8,9,10].

The antibacterial, antifungal and antiviral potential of nanoparticles is connected to their small size, high surface-to-volume ratio, surface charge and the generation of reactive oxygen species (ROS). The small size of the nanoparticles allows them to penetrate bacterial cell membranes through absorption and diffusion mechanisms. In the case of Gram-negative bacteria, the nanoparticles penetrate the negatively charged outer thin peptidoglycan layer (7–8 nm) by electrostatically interacting with it and generating ROS, which damages the cells and inhibits bacteria [11]. Also, the slight negative charge of the Gram-positive bacteria membrane allows the negatively charged superoxide radicals and peroxide generated by nanoparticles to penetrate the inside of bacterial cells and denature proteins or interact with DNA [12]. Other references show that copper nanoparticles bind to carboxyl or amine groups of cell membranes and penetrate inside the bacterial cell [13]. The other mechanism of copper nanoparticles antimicrobial action is attributed to the damage of the cell membrane and protein denaturation [14].

The antifungal properties of TiO_2_ nanoparticles are attributed to the inhibition of the film formation [15]; meanwhile, the mechanism of other nanoparticles antifungal activity is related to the damage induced by ROS on proteins and DNA of the cells [16].

The ability of small silver nanoparticles to attach to virus particles and inhibit their connection to host cells explains their antiviral properties against Monkeypox and HIV1 viruses [17,18]. Graphene oxide and silver nanocomposites exhibit antiviral activity against both enveloped and non-enveloped viruses due to their interaction with lipid membranes and spike proteins, which disturbs host cell attachment [19,20]. The mechanisms by which nanoparticles, nano oxides and nanocomposites act against microbes are not fully understood and require further study [21].

Gamma irradiation has also been investigated to refine nanoparticle architecture, enhance ROS generation, and improve antimicrobial function [22,23,24]. Despite promising results in standalone systems (e.g., Ag–TiO_2_, CuO–TiO_2_, or GO–TiO_2_), the optimal combination of TiO_2_, Ag/Cu, GO, and gamma radiation treatment for enhanced leather antimicrobial coatings remains poorly explored [2,6,10,23]. There are diverging hypotheses: some assert that graphene oxide enhances both dispersion and ROS production, while others observe reduced activity due to site blockage on TiO_2_ [9,10]. Gamma irradiation may further modulate material structure, providing activation benefits beyond traditional synthesis routes [22,24].

This study aims to investigate the effects of coating leather surfaces with TiO_2_ nanoparticles decorated with Ag, Cu_2_O/CuO, and graphene oxide (GO), followed by controlled gamma irradiation. This topic is not covered in the literature. Investigations were performed using SEM/EDS, ATR/FTIR, a color photodegradation test and leather fastness resistance standard methods. These allowed highlighting the influence of gamma irradiation on the antimicrobial and self-cleaning properties of new nanocomposites embedded in finishing films. By integrating multifunctional nanomaterials and gamma radiation treatment, this study proposes a novel, scalable platform for the next generation of durable, long-lasting antimicrobial leather with potential applications in healthcare and consumer goods.

## 2. Materials and Methods

### 2.1. Composite Nanoparticles Preparation

**Figure 1:**
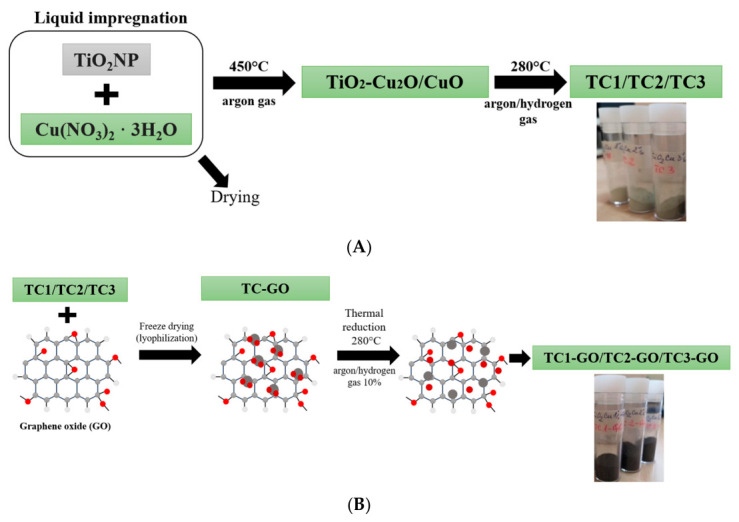

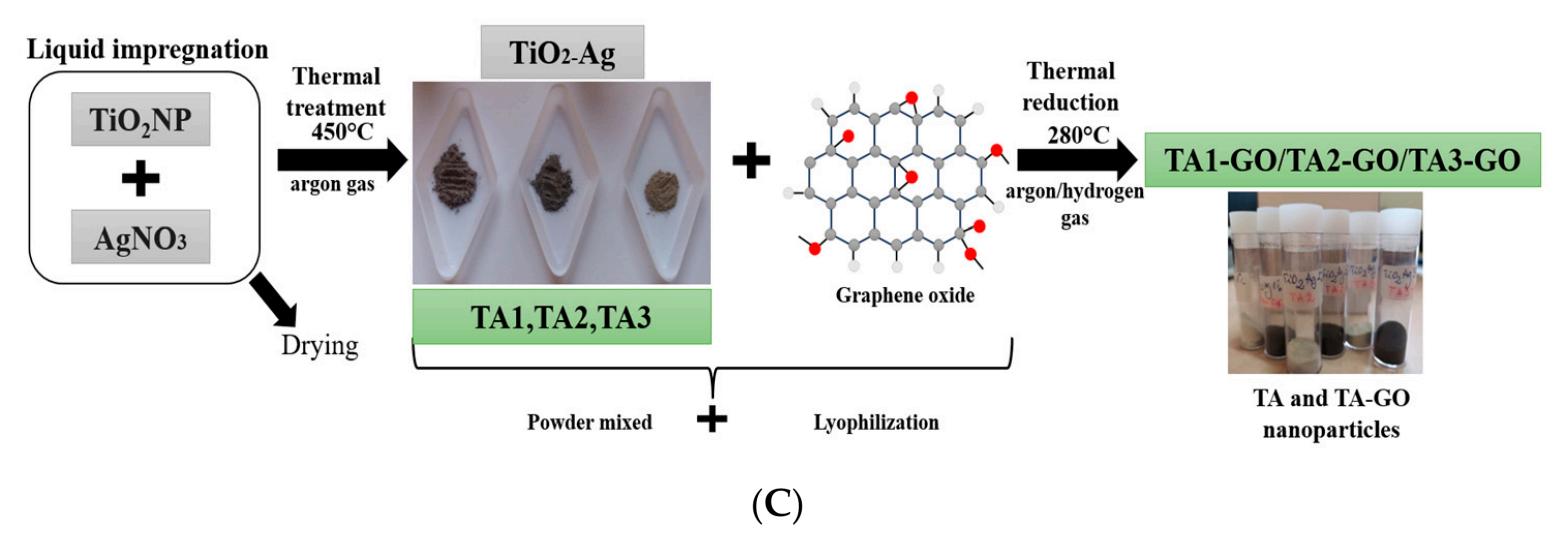
Nanocomposites preparation scheme: (**A**) TiO_2_ decorated with Cu_2_O/CuO (TC1, TC2, TC3); (**B**) TiO_2_ decorated with Cu_2_O/CuO-GO (TC1-GO, TC2-GO, TC3-GO); (**C**) TiO_2_ decorated with Ag (TA1, TA2, TA3) and Ag-GO (TA1-GO, TA2-GO, TA3-GO).

Two types of composites based on TiO_2_ nanoparticles (NPs) decorated with Cu_2_O/CuO or Ag and graphene oxide (GO) were prepared and characterized according to the methods presented in the previous publications [25,26,27]. Figure 1 shows the synthetic scheme of nanocomposite preparation. In brief, the nanocomposites were prepared using the liquid impregnation method with TiO_2_ NPs (5 nm anatase TiO_2_A-110 from GetNano, Saint-Cannat, France), as well as Cu_2_O/CuO and Ag nanoparticles, in a variety of products. Graphene oxide (GO), which was prepared by oxidizing graphite flakes (Merck KGaA, Darmstadt, Germany) with H_2_O_2_ according to Hummer’s method [28,29], was added to another range of products. The liquid impregnation method involves stirring TiO_2_ powder in copper(II) nitrate trihydrate, followed by evaporation and calcination in an argon atmosphere at 450 °C and in an argon/hydrogen atmosphere at 280 °C. The successive processes of dispersing in double-distilled water and freeze-drying aim to prepare a homogeneous powder. TiO_2_ NPs decorated with Cu_2_O/CuO were denoted TC1, TC2, and TC3 (Figure 1A), corresponding to 1%, 2%, and 3% Cu concentrations, respectively [27]. TC1, TC2, and TC3 powders were mixed with GO powder at a 10:1 weight ratio and then thermally treated at 280 °C for 90 min in an argon/hydrogen atmosphere. This was followed by lyophilization. The resulting products were labeled TC1-GO, TC2-GO, and TC3-GO (Figure 1B). Similarly, TiO_2_ NPs decorated with Ag were prepared by mixing TiO_2_ NPs with different concentrations of AgNO_3_ corresponding to 1%, 2%, and 3% Ag by the weight of TiO_2_ NPs. This was followed by thermal treatment at 450 °C for 150 min in an argon atmosphere. The products TA1, TA2, and TA3 were ground into a powder (Figure 1C). TA1-GO, TA2-GO, and TA3-GO were prepared by mixing TA1, TA2, or TA3 with GO at a weight ratio of 10:1. This was followed by thermal treatment in an argon/hydrogen atmosphere at 280 °C for 90 min, followed by lyophilization (Figure 1C).

### 2.2. Leather Coating with Composite Nanoparticles and Gamma Radiation Treatment

**Figure 2:**
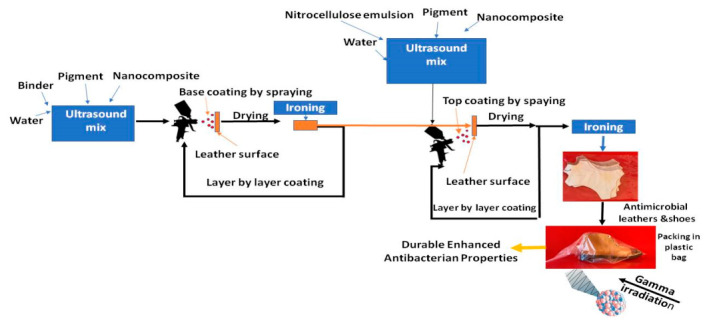
The scheme of leather coating with nanocomposites and the treatment using gamma irradiation for durable antimicrobial properties.

Figure 2 shows the process of treating the leather and how gamma irradiation treatment may enhance its antimicrobial properties.

The crust sheep leathers, processed in the Leather Research Department of ICPI, were coated by spraying a blend of base-coat finishing polymers (anionic acrylic resins and fillers in a water emulsion with 17–19% solids and a pH of 7–9) mixed with white casein-base pigment (60% solids and a pH of 9) and composite nanoparticles. The coating was then fixed with an emulsion of nitrocellulose lacquer (an anionic, water-based nitrocellulose with 14.5–17.5% solids), supplied by SC Triderma SRL (Bucharest, Romania). The concentration of composite nanoparticles was determined based on previous research trials [Patent Application A00572 from 2021], which were conducted to achieve antimicrobial properties. The coating technology used for coating all leather samples is common in industrial leather finishing and is presented in the Supplementary Materials. The control samples were made with the same technology, without nanocomposites.

The coated leathers were vacuum packed in plastic bags, sealed with a heat sealer, and transferred to a ^60^Co gamma installation at the IFIN-HH, IRASM Radiation Processing Center, a Gamma Chamber 5000 self-shielded irradiator (BRIT, Navi Mumbai, India), for treatment at 25 kGy [30,31]. The absorbed dose was measured using a calibrated ethanol-chlorobenzene (ECB) dosimetry system [32]. The ECB dosimeters were measured using the oscillometric method (Radelkis reader, Institute of Isotopes, Budapest, Hungary). Irradiation was performed in cylindrical geometry with a minimum dose rate of 4.9 kGy/h. The measurement uncertainty of the absorbed dose was 3.3%, and the dose uniformity ratio (the ratio of the maximum to minimum absorbed doses) was 1.26.

### 2.3. Leather Surface Characterization and Functionality Assessment

The leather surface was characterized for its main fastness resistance properties to determine the influence of nanocomposites on fastness resistances, color difference, surface morphology, and composition. Abrasion fastness was assessed using the method described in SR EN 13520:2003 [33], using a Martindale device (James Heal, Halifax, UK). The other characteristics were tested using a rubbing test in dry, wet or artificial perspiration conditions, according to SR EN 11640:2013 [34], with the Giuliani Rub Tester (Turin, Italy), as well as a water drop resistance test according to SR EN ISO 15700:2000 [35]. The results were expressed as marks from 1 to 5; 5 represented the best resistance when the color was compared with the initial color using grayscale. The first mark indicated the color change in the leather, and the second mark the color change in materials used for abrasion or rubbing. In the case of the water drop test, the mark represents the color change in the leather after its interaction with a water drop and in a dry state.

The color difference caused by the nanocomposites on the leather surface and gamma irradiation treatment was measured with a X-Rite Ci7600 (400) benchtop Spectrometer equipped with a Color iQC Basic Perpetual License Version v10.7.0.38 (Grand Rapids, MI, USA). The leather surface morphology and composition were analyzed using a FEI Quanta 200 scanning electron microscope (FEI, Eindhoven, The Netherlands) equipped with an EDX (Ametek EDAX Element, Mahwah, NJ, USA). Changes induced by gamma irradiation on surface chemical groups were assessed using a Bruker Vertex 70 instrument (Bruker, Ettinger, Germany) with the following working parameters: 4 cm^−1^ resolution, 0.1 cm^−1^ wavenumber accuracy, 0.1% T photometric accuracy and 32 scans in the 4000–400 cm^−1^ spectral range. The spectra were vector normalized and baseline corrected with the OPUS 7.5 software.

#### 2.3.1. Antimicrobial Leather Surface Activity

Antimicrobial determinations were performed according to the standard EN ISO 16187:2013 [36].

The tested strains were *Escherichia coli* ATCC 25922 and *Staphylococcus aureus* ATCC 6538, which were supplied by Mediclim, Otopeni, Romania.

The inoculum was prepared by transferring one colony from the stock culture of each strain into 20 mL of nutrient broth (NB) or tryptic soy broth (TSB). The mixture was then incubated in a shaking incubator at 37 ± 2 °C for 16 h, after which the number of bacteria was estimated by microscopy. Then, a physiological saline solution containing 1% NB or TSB was prepared as a medium for the bacterial suspension, which had a concentration of 1.0 × 10^5^ CFU/mL. The addition of physiological saline solution (0.85–0.9% NaCl) with 1% NB or TSB serves to maintain bacterial viability and suspension stability prior to inoculation. This stability ensures that the initial inoculum density remains consistent, which is crucial for the accuracy and reproducibility of minimum inhibitory concentration (MIC) and kill-time studies. This solution was used as the test inoculum. Briefly, the test samples were placed in Petri dishes, and 0.2 mL of the inoculum was pipetted onto different points on the surface of each sample. Then, each sample was covered with a piece of film. Immediately after inoculation, 10 mL of physiological serum was added, and the samples were incubated at 37 °C for 24 h. The number of viable bacteria was determined using the plate counting method. The results are the averages of three determinations and are expressed as CFU/mL (SI). The bacterial load reduction percentage (R%) was calculated based on the average CFU concentration values using Formula (1):

(1) $$R\%=\frac{IC_{0\;}-IC_{24\;}}{IC_{0\;}}\;\times \;100$$

where:

R% is the bacterial load reduction;
IC_0_ is the average value of the initial inoculum concentration; and
IC_24_ is the average value of the inoculum concentration after 24 h of interaction with leather samples.

#### 2.3.2. Photo Decomposition of Organic Stains

The decomposition of organic stains under visible light exposure was simulated according to the protocols published in the literature [37,38,39]. Briefly, a 30 μL drop of a 300 mg/L methylene blue (MB) water solution was pipetted onto leather surfaces and left to dry naturally. The stained leather samples were exposed to visible light using a 500 kW lamp (NXS-500 P) with an average intensity of 8 mW/cm^2^ from a distance of 20 cm for 60, 120 and 240 min, with 30 min breaks so that the sample did not heat up and resume exposure. Color parameters were recorded using a Ci7600 benchtop spectrophotometer equipped with a ColoriQC Basic Perpetual Licence (Grand Rapids, MI, USA). The initial methylene blue stain color served as the reference, and color differences were recorded for three intervals as an average of three measurements.

### 2.4. Statistical Analyses

The experiments were performed in triplicate, and the results were expressed as mean values with standard deviations (SDs). ANOVA in Microsoft Excel 2019 was used to analyze the biological results for values of the *p* coefficient less than 0.05 (statistically significant difference between samples and controls). The average values for microbial load measurements and standard deviations are presented in the Supplementary Materials. The reduction percents were calculated using the average values of bacterial loads according to Formula (1) and the standard specifications [36].

## 3. Results and Discussion

### 3.1. Leather Coated with Nanoparticle Composites Based on TiO_2_ Decorated with Ag, Cu_2_O/CuO and Graphene Oxide

**Figure 3:**
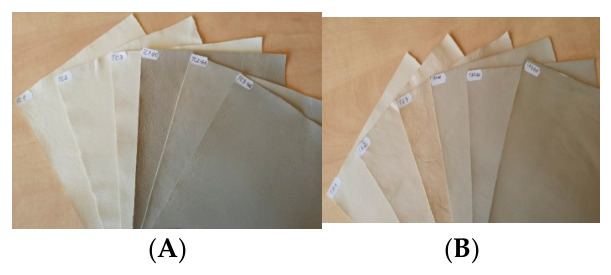
Leathers coated with TiO_2_ NPs decorated with Cu_2_O/CuO and Cu_2_O/CuO/GO (**A**); Leathers coated with TiO_2_ NP decorated with Ag and Ag/GO (**B**).

The influence of decorating component concentrations on surface finesse and color modification in leathers coated with nanocomposites was compared, with and without graphene oxide embedded in film-forming polymers. Figure 3A,B illustrate leather surfaces resembling traditional leathers when nano finishing coats do not contain graphene oxides.

Meanwhile, the color of leathers coated with nanocomposites containing graphene oxide changed substantially from white to medium and dark gray. This color change suggests using these nanocomposites to finish medium- and dark-colored leather goods. Since leather articles are primarily medium and dark in color, multifunctional leather articles will find a good market among enthusiasts of exclusive, techno, or smart goods.

Leather coated with multiwall carbon nanotubes [40] may only be used for black colors. It allows new properties to be added to the leather surface, such as electric conductivity (touch screen function), antimicrobial properties against Gram-positive and Gram-negative bacteria, and self-cleaning properties against greasy dirt. In another published study [41], a flower-like Ag-TiO_2_-SiO_2_ nanocomposite for leather coating with fluorescent, antimicrobial and hydrophobic properties was presented as a color shield against fading. The yellow/brownish color of the leather surface was imparted by the silver content of the composite nanoparticles.

Table 1 shows the lightness and the total color differences values for the leather surfaces before and after gamma irradiation. The colors generated by the photo colorimeter can be seen in the Supplementary Materials (SI).

The lightness difference of TA-GO and TC-GO leathers shifted to higher values after gamma irradiation, suggesting an influence of the graphene oxide component on color discoloration. ΔL* was 4.07–6.95 and 1.49–2.05 for composites with GO, compared with −1.27–0.19 and −0.37–0.64 for composites without GO.

Gamma irradiation had a greater influence on TA-GO (ΔE of 5.07–7.43)-coated leathers than on TC-GO (ΔE of 2.22–3.37) samples.

Color analyses showed that the leather coated with nanocomposites containing graphene oxide and silver are most affected by gamma irradiation. This behavior can be attributed to the oxidizing properties of silver nanoparticles [1,42] and the presence of graphene oxide, which is activated by gamma irradiation. This leads to an enhanced production of oxygen species, resulting in leather discoloration.

### 3.2. Fastness Resistance of Leather Surfaces Coated with Nanoparticle Composites Based on TiO_2_ Decorated with Ag and Cu_2_O/CuO and Graphene Oxide

Table 2 shows the fastness resistances of leather surfaces coated with composite nanoparticles. It can be seen that the nanoparticles improve the abrasion resistance of samples TA2, TA3 and TC3. The presence of graphene oxide substantially increases the abrasion resistance for samples TA1-GO, TA2-GO, TC1-GO and TC2-GO. The presence of carbon derivates was shown to increase the abrasion resistance of a leather surface from 38,400 cycles to 51,200 cycles [40]. This is similar to the samples coated with TA2-GO, TA3-GO, TC2-GO and TC3-GO, but very different from a leather surface coated with Ag-TiO_2_-SiO_2_ nanocomposites, which has an abrasion resistance of 3000 cycles [41]. Wet rubbing resistance was significantly improved for samples coated with TA2-GO, TA3, TA3-GO, TC2-GO, and TC3-GO. These values were superior to those of a leather surface coated with ZnO nanoparticles, as reported by other authors, compared to control samples, where the value increased from 1 to 2 [43].

A significant difference was recorded for perspiration rubbing resistance compared to the control sample, suggesting that new finishing coats may enhance leather functionality and comfort.

### 3.3. Antimicrobial Activity of Leather Surfaces Before and After Gamma Radiation Treatment

The antimicrobial activity of leather surfaces against Gram-positive and Gram-negative bacterial strains is slightly enhanced after gamma irradiation and is increased after 60 days of gamma irradiation, as can be seen in Table 3, Table 4, Table 5 and Table 6 and Supplementary Materials (SI). Control samples show an obvious reduction in microbial load after gamma irradiation, but this resistance is not durable. After 60 days, resistance substantially decreased from 97.66–97.80% to 36.50–40.00%.

While increased antimicrobial properties have been reported for leather coated with nanoparticle composites [44,45,46,47], enhanced antimicrobial activity after 60 days of treatment with 25 kGy gamma radiation has not yet been reported. Similar reductions in bacterial load (100%, *p* < 0.05) were recorded when bovine leathers or pig skins were treated with ZnO/TiO_2_ NPs [45] or Ag NPs [46]. The main differences from the process presented in this manuscript are the reduced potential for transfer to an industrial environment due to the high consumption of nanomaterials in immersion processes and the generation of significant quantities of effluents.

Gamma irradiation modifies materials based on ionizing radiation properties, producing free radicals, bonds breaking, crosslinking, atom dislocations, and vacancies when it interacts with matter. Free radicals generated by gamma irradiation were found to be centered on the oxygen of proteins via EPR signal analysis over time [48]. In the case of solid complex materials, the radiolysis products can be trapped inside the collagen structure of leather or polymeric components of finishing coats and produce further changes in time under various environmental conditions. Similar effects were reported after 7.5 months of polyethylene treatment with 20 kGy gamma radiation when peroxyl radicals were found to be released and to scissor the chemical structure, releasing carbonyl, hydroperoxides and ketones [49].

It is reasonable to suppose that gamma irradiation induces complex processes that influence not only nano titanium dioxide decorated with Ag, Cu_2_O/CuO and GO, but also the micro titanium dioxide pigment used in the base-coat layer, which has the potential to slightly enhance the antibacterial effect over 60 days. We observed a slight enhancement in microbial load reduction, from 12% in the initial stage to 36% after 60 days, in the control samples as well. The nano titanium dioxide composites embedded in film-forming polymers and gamma irradiated could modulate the materials’ electronic and photocatalytic activity over time by bandgap decreasing. The reduced band energy gaps of TA, TA-GO, TA-GR (reduced graphene), and TA-GT (graphene) compared to TiO_2_ NPs range from 3.27 eV to 3.09, 3.06, 3.04, and 3.05 eV, as reported [30].

### 3.4. Morphology and Composition Analyses by SEM/EDS

**Figure 4:**
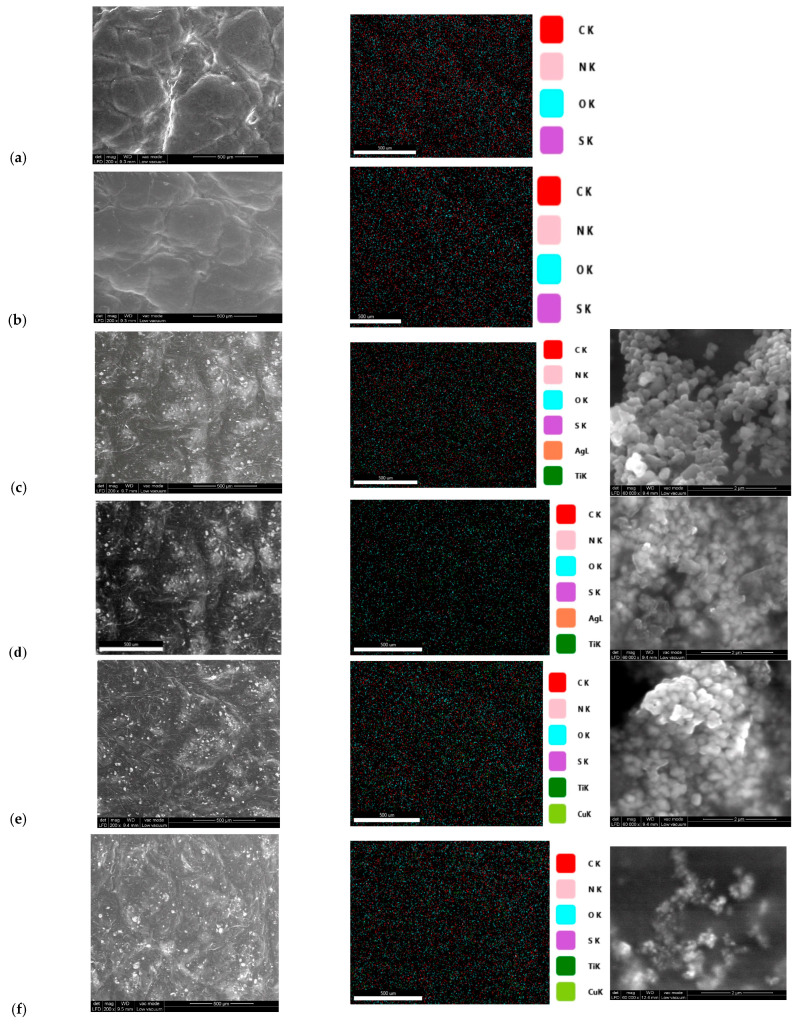

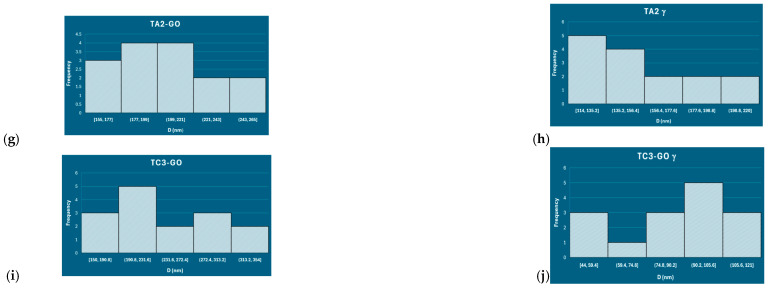
SEM images with elemental composition (EDS) for the control leather surface: before (**a**) and after γ irradiation (**b**) (200×); coated with TA2-GO, before (**c**) and after γ irradiation (**d**) (200×, 60,000×); and coated with TC3-GO, before (**e**) and after γ (gamma) irradiation (**f**) (200×, 60,000×). The particle size distribution: TA2-GO before (**g**) and after γ irradiation and (**h**) TC3-GO before (**i**) and after γ irradiation (**j**).

The leather surface was analyzed before and after gamma irradiation treatment. The control leather surfaces were very similar before and after gamma irradiation (Figure 4a,b), exhibiting a composition typical of collagen-based materials (C, N, O, S). Similar conclusions were found after the conservation of proteins [36] or vegetable-tanned leathers [50] with a 25 kGy gamma irradiation dose. Figure 4c–f and Supplementary Materials present the morphology of the leather surface with nanoparticle composites before and after gamma irradiation. The uniform deposition of nanocomposites on the leather surface correlates well with good antimicrobial resistance. Elemental composition analysis revealed the presence of Ag and Cu on the leather surfaces. Analysis of nanoparticle composite agglomerates at 60,000 magnifications showed that they are composed of nanosized particles. Further studying the morphology of the nanoparticle at higher magnifications, at 120,000×, revealed slightly smaller particle sizes on leather surfaces treated with gamma irradiation. The smaller size of nanoparticle composites explains the larger surface area available for ROS generation in the interaction with UV [28] or visible light, resulting in improved photoreactivity. Similar effects of gamma irradiation on boron carbide microparticles at high doses have been demonstrated [51], with the microparticle size decreasing from 19.87 ± 4.84 μm to 14.66 ± 1.57 μm. In our study, the particle sizes decreased from 204.00 ± 32.00 nm for the unirradiated TA2-GO composite to 154.00 ± 34.00 nm for irradiated nanocomposites from the leather surface (Figure 4g,h). For TA3-GO nanocomposites, irradiated particle agglomerates decreased from 242.00 ± 59.00 nm to 85.00 ± 22.00 nm (Figure 4i,j). Similar results were recorded for the other nanocomposites on leather surfaces, as shown in the Supplementary Materials (SI). The standard deviations of the sizes of the nanocomposite determined in this study were within the limits found by other researchers for a larger number of samples analyzed by SEM image analysis [52,53,54].

### 3.5. Photocatalytic Decomposition of Organic Stains Under Visible Light

**Figure 5:**
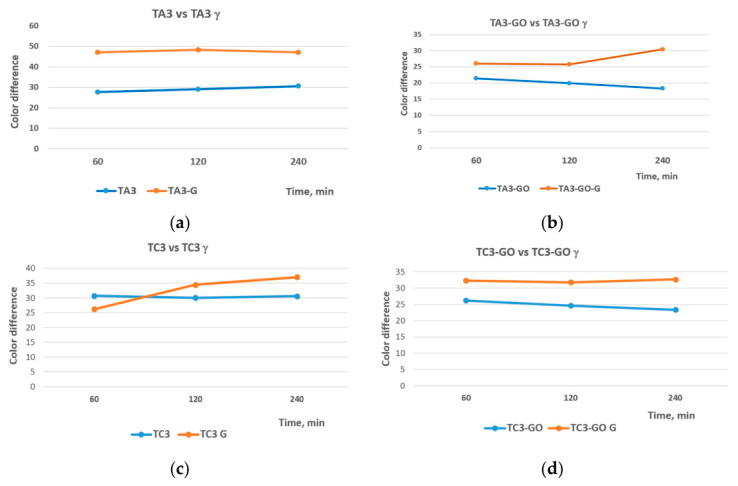
Color differences of MB stain exposed to Vis light and measured for irradiated and unirradiated leather surfaces coated with (**a**) TA3, (**b**) TA3-GO, (**c**) TC3 and (**d**) TC3-GO nanocomposites (G is the symbol for gamma (γ)-irradiated samples: TA3G, TA3-GO G, TC3 G, TC3-GO G).

In most cases, the photocatalytic activity of TiO_2_ nanoparticles (NPs) decorated with Ag, Cu_2_O/CuO, and graphene oxide (GO) increased activity after irradiation with 25 kGy gamma rays. The methylene blue stain color difference, which expresses the color degradation, was higher for irradiated samples than for unirradiated samples. Figure 5a–d show the best performances of the irradiated samples compared to the unirradiated samples: TA3/TC3 versus gamma-irradiated TA3/TC3 and TA3-GO/TC3-GO versus gamma-irradiated samples, respectively. The Supplementary Materials (SI) presents the photocatalytic behavior of all analyzed samples. TA1/TC1 and TA1-GO/TC1-GO samples clearly show enhanced reactivity after gamma irradiation. Meanwhile, TC2, TA2 and TA2-GO samples behave as if the gamma radiation does not activate the photocatalytic response of titanium dioxide nanoparticles to visible light. The absence of a photocatalytic response when exposed to gamma radiation, particularly for products containing 2% Ag or Cu/Cu_2_O, may be due to agglomerations hindering the active centers of TiO_2_ NPs. In the case of the TA2-GO sample, another hypothesis is that oxidative species are forming quickly, resulting in discoloration of the coating and the formation of compounds that suppress the activity of TiO_2_ NPs. This hypothesis aligns with the high values recorded for ΔL* and ΔE* (Table 1) for this sample, which exhibited the greatest discoloration. Further investigation is needed to clarify this. The highest photocatalytic activity induced by gamma irradiation was recorded for the TA3 γ-irradiated sample (Figure 5a) after 120 min, when the color difference between the initial methylene blue stain and the exposed stain was 48.35. Figure 5b,d show enhanced photoactivity of leather surfaces coated with GO-containing nanocomposites and gamma-irradiated samples compared to unirradiated samples.

We can conclude that gamma irradiation induced higher photocatalytic reactivity with a long-lasting effect.

The enhanced photocatalytic and antimicrobial properties of decorated nano titanium dioxide may be explained by the decreased electron–hole pair recombination rate due to the presence of Ag, Cu_2_O/CuO, and GO, as well as the increased surface area under gamma irradiation. This behavior aligns with the antimicrobial properties and supports the hypothesis of ROS generation and its oxidative effects on the decomposition of organic surface loads. As shown in other studies of decorated titanium dioxide nanoparticles, the Fermi levels of Ag [55,56], Cu_2_O/CuO and GO are lower than those of titanium dioxide nanoparticles. The charge transfer between TiO_2_ and the decorating components (Ag, Cu_2_O/CuO, GO) needs further investigations. The presence of graphene oxide, silver, or copper oxides can greatly reduce the electron–hole recombination rate, resulting in a stronger photocatalytic reaction that, over time, may be augmented by gamma radiation. The authors studying previously the TC-GO [28] and TA-GO [57] nanocomposites using voltametric methods revealed improved electrocatalytic and redox activity due to the electron transfer facilitated by graphene oxide. Similar studies could not be performed in the same conditions as for pristine nanocomposites due to the complex matrix of nanocomposites embedded in film-polymers and deposited on the leather surface. The reduction of the band gap of pristine variously decorated TiO_2_ nanocomposites, from 3.27 eV to 3.04 eV, was reported [30], as well as the decomposition of amaranth dye as an effect of oxidative species generation under UV and Vis light exposure [27]. The presence of graphene was found to enhance the photoactivity of TiO_2_-Ag composite [27].

Color measurements on leather coated with nanocomposites showed that gamma irradiation enhanced the photoreactivity of the irradiated samples compared to the unirradiated samples (Figure 5 of above and Figure S3 of the Supplementary Materials). Thus, the indirect effects of modified titanium dioxide nanoparticles embedded in an organic polymer matrix, deposited on the leather surface and gamma irradiated are presented.

**Figure 6:**
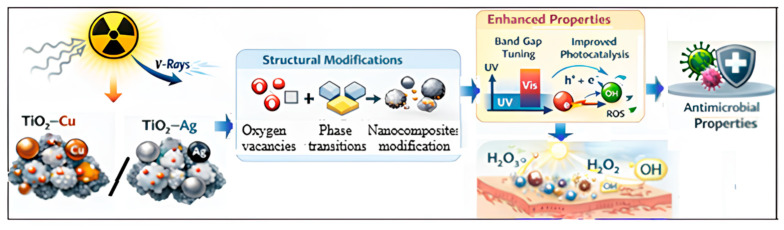
Schematic illustration of the proposed mechanism for antimicrobial and photocatalytic activity of leathers coated with TiO_2_ NPs decorated with Ag, Cu_2_O/CuO, and GO and gamma irradiated.

Figure 6 shows the potential mechanism of the long-lasting antimicrobial and enhanced photoreactive properties of the prepared leather surface. The hypothesis is that gamma irradiation induces crystal defects in the nanoparticles, generating defects, atom dislocations, oxygen vacancies, and phase transitions that diminish the band gap energy and enhance the generation of reactive oxygen species.

### 3.6. ATR/FTIR Analyses of Leather Surface

FTIR spectroscopy enables the rapid identification of materials composition and structure modifications resulting from various chemical or physical treatments, such as plasma, gamma irradiation, laser ablation, and mechanosynthesis [58,59].

#### 3.6.1. FTIR Results

**Figure 7:**
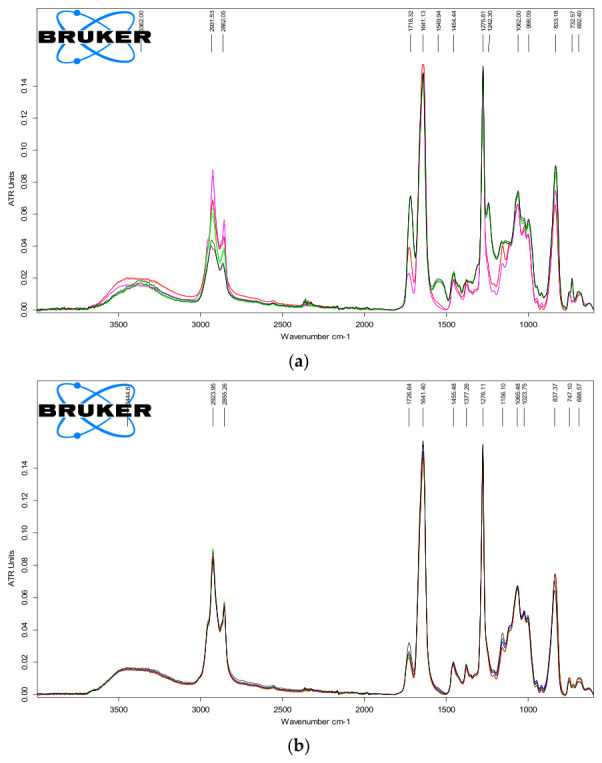

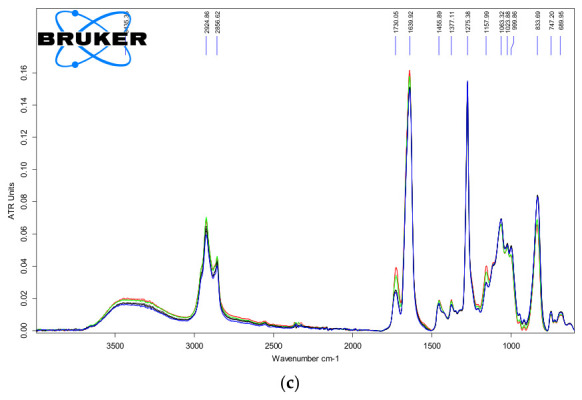
ATR-FTIR spectra of leather samples before and after gamma irradiation: (**a**) control, control irradiated at 25 kGy, TA1, TC1; (**b**) TA1-GO, TA1, irradiated TA1-GO, irradiated TA1; (**c**) TC1-GO, TC1, irradiated TC1-GO, irradiated TC1.

Figure 7a–c show the ATR-FTIR spectra of the analyzed leather samples before and after irradiation.

#### 3.6.2. Discussion of Chemical Structure of Various Leather Coatings Pre and Post Gamma Irradiation

The spectra show similar characteristics. As may be seen from Figure 7a–c, the leather samples treated with nanocomposites hinder the Amide II collagen band of 1550 cm^−1^ of leather as compared to the control samples. In the spectra are visible small differences of band shape, intensity and position between samples in the region of OH (3441 cm^−1^), CH (2925 cm^−1^), C=O (1730 cm^−1^), and C-O (1063 cm^−1^) stretching bands. The changes are correlated with fine compositional and structural differences that modify samples’ reactivity and stability. The shift of the OH and CO bands position indicates modification of the hydrogen bonding pattern. TheTiO_2_ band is visible at about 671 cm^−1^ [60].

The samples with the GO component exhibit more intense OH stretching and bending vibrations at 3441 and 1639 cm^−1^ (0.039 ATR units as compared to 0.025 ATR units for TC-GO and TC samples for the OH bending peak), attributed either to hydroxyl groups of GO or adsorbed water molecules, associated with higher hydrophilicity. TC1 samples have more OH groups (0.018 ATR units) and C=O groups (0.039 ATR units) as compared to TA1 samples with 0.014 and 0.022 ATR units, respectively. Irradiated samples show fewer OH groups (0.014 ATR units), fewer C=O groups (0.024 ATR units) and more CH groups (0.062 ATR units) as compared to samples with 0.015, 0.025, and 0.056 ATR units, respectively, for the case of irradiated TC1-GO and unirradiated TC1-GO.

#### 3.6.3. Chemical Changes Link to Photocatalytic/Antimicrobial Activity, and Practical Implications

The interplay between the small compositional variation emphasized by FTIR and the surface morphology evidenced by SEM analysis finely modulates the photo reactivity and the antimicrobial properties of these multicomponent materials. The physical chemical characterization demonstrated that the presence of nanocomposites, the application technology and the gamma irradiation treatment allow the leather’s integrity to be preserved while providing durable antimicrobial properties. Further investigations will be devoted to understanding the mechanism of the long-lasting reactivity of leather surfaces coated with nanocomposites with and without graphene oxide after gamma irradiation. Indirect analyses, like antibacterial properties against Gram-positive and Gram-negative strains, photodegradation of organic stains as an effect of ROS generation under visible light exposure and the improved fastness resistances, showed the applicative potential of new coatings for more durable and protective materials against environment aggressions. The impact of new coatings on human health will be evaluated by wearing and biocompatibility tests. Gamma irradiation of footwear leather coated with nanocomposites showed good potential for long-lasting antimicrobial properties, with an application in healthcare. These results provide a solution for antimicrobial treatment of medical equipment, including footwear, with potential applications in other areas such as furniture, aircraft or car upholstery, clothing and bags.

## 4. Conclusions

This research aimed to create leather surfaces with long-lasting antimicrobial properties by using TiO_2_-based nanocomposites decorated with silver (Ag), copper oxides (Cu_2_O/CuO) and graphene oxide. The nanoparticles were applied to the leather surface in finishing films and subsequently activated by gamma irradiation at 25 kGy.

After 60 days, not only was the antibacterial activity maintained compared to the initial state, it also increased slightly compared with unirradiated leathers. The bacterial load on the leather surface decreased from 2.19 × 10^3^ ± 2.51 CFU/mL to 0 (TA3-GO sample). The nanocomposites also improved photocatalysis under visible light, promoting self-cleaning by degrading methylene blue stains. Compared to classical samples, the nanocomposites with graphene oxide increased the fastness to abrasion, wetness and perspiration rubbing. SEM/EDS analyses confirmed the uniform dispersion of the nanocomposite on the leather surface, as well as the particle sizes, which ranged from 77 to 242 nm. ATR/FTIR spectroscopy investigations revealed minor structural differences between irradiated and unirradiated samples, and confirmed the integrity of the leather finishing. Gamma irradiation significantly influenced the color of the TA-GO range of samples, with color differences ranging from 5.07 to 7.43.

This proposed methodology is compatible with industrial leather finishing and demonstrates the feasibility of producing antimicrobial-protected leather, which has potential applications in biosafety products.

Compared to previous research, the application of these nanocomposites makes it possible to obtain enhanced photocatalytic effects and long-lasting antimicrobial properties after gamma irradiation treatment.

Further research will focus on the toxicological impact of using nanocomposites as an advanced leather coating through wear simulation, determining leaching of the nanocomposites, evaluating cytotoxicity limits, and assessing biocompatibility.

## 5. Patents

OSIM Patent Application A00572 from 2021, “Piei cu proprietati antimicrobiene durabile si procedeu de realizare a acestora” (“Leather with durable antimicrobial properties and process for the production therefore”), Gaidau C.; Stanca M.; Stanculescu, I.R.; Rosu, M.C., Socaci, C.A.; Alexe, C.A.; Constantinescu, R.R.

## Figures and Tables

**Table 1:** Lightness and total color differences of leather surfaces coated with nanocomposites before and after gamma irradiation.

γ-Treated Leather Sample/Initial Leather Sample	ΔL*	ΔE*
TA1-γ/TA1	−1.27	3.47
TA2-γ/TA2	−0.77	1.12
TA3-γ/TA3	0.19	4.43
TA1-GO-γ/TA1-GO	4.07	5.07
TA2-GO-γ/TA2-GO	6.95	7.43
TA3-GO-γ/TA3-GO	5.11	5.92
TC1-γ/TC1	−0.37	2.42
TC2-γ/TC2	0.64	1.11
TC3-γ/TC3	−0.26	2.54
TC1-GO-γ/TC1-GO	2.05	2.46
TC2-GO-γ/TC2-GO	1.84	3.37
TC3-GO-γ/TC3-GO	1.49	2.22

**Table 2:** Fastness resistances of leather surfaces coated with composite nanoparticles as compared to control sample.

Sample	Abrasion Resistance, Number of Cycles	Rubbing Resistance, Marks				Water Drop Resistance
		Dry 50 Cycles	Dry 100 Cycles	Wet 50 Cycles	Perspiration 50 Cycles	Marks 1–5
TA1	17,600	5/5	4–5/4–5	1–2/1	1/1	1
TA1-GO	49,600	5/5	4–5/4–5	1–2/2	1/1	1
TA2	38,000	5/5	4–5/4–5	1–2/1	1/1	2
TA2-GO	51,200	5/4–5	4–5/4	3/3–4	2/2	4–5
TA3	38,400	5/5	4–5/4–5	3/3–4	1/1	2–3
TA3-GO	51,200	5/5	4–5/4–5	3/4	2/3	4–5
TC1	17,600	5/5	3–4/3	1/1	1/1	2
TC1-GO	49,000	5/5	3–4/4	1–2/2	1/2	2–3
TC2	26,000	5/5	5/5	2/2	1–2/1	4
TC2-GO	51,200	4/4	3–4/3	3/4–5	3/4	5
TC3	38,000	5/5	5/5	3/3	1–2/2	4–5
TC3-GO	51,200	5/5	5/5	3/4–5	3/4–5	5
Control	38,400	5/5	5/4–5	1/1	1/1	2–3

**Table 3:** Reduction of the microbiological load with *Escherichia coli* ATCC 25922 (1.00 × 10^5^ CFU/mL) of unirradiated leathers, after irradiation and 60 days afterwards, TA and TA-GO samples, compared to control sample.

Sample	Leather Finished with Nanocomposites and Unirradiated	Leather Finished with Nanocomposites and Irradiated	Leather Finished with Nanocomposites and Irradiated, After 60 Days
	Reduction, %	Reduction, %	Reduction, %
TA1	95.55	99.83	99.96
TA2	94.70	99.91	100
TA3	97.76	99.95	100
TA1-GO	97.47	99.96	100
TA2-GO	97.60	99.96	100
TA3-GO	97.76	99.95	100
Control	12.90	97.80	36.50

**Table 4:** Reduction of the microbiological load with *Staphylococcus aureus* ATCC 6538 (1.00 × 10^5^ CFU/mL) of unirradiated leathers, after irradiation and 60 days afterwards, TA and TA-GO samples, as compared to control sample.

Sample	Leather Finished with Nanocomposites and Unirradiated	Leather Finished with Nanocomposites and Irradiated	Leather Finished with Nanocomposites and Irradiated, After 60 Days
	Reduction, %	Reduction, %	Reduction, %
TA1	98.98	99.82	99.91
TA2	99.08	99.92	100
TA3	99.09	99.95	100
TA1-GO	99.15	99.79	100
TA2-GO	97.86	99.85	100
TA3-GO	90.86	99.96	100
Control	17.65	97.66	40.00

**Table 5:** Reduction of the microbiological load with *Escherichia coli* ATCC 25922 (1.00 × 10^5^ CFU/mL) of unirradiated leathers, after irradiation and 60 days afterwards, TC and TC-GO samples, compared to control samples.

Sample	Leather Finished with Nanocomposites and Unirradiated	Leather Finished with Nanocomposites and Irradiated	Leather Finished with Nanocomposites and Irradiated, After 60 Days
	Reduction, %	Reduction, %	Reduction, %
TC1	98.43	99.88	100
TC2	91.95	99.94	100
TC3	99.02	99.93	100
TC1-GO	95.44	99.93	100
TC2-GO	92.70	99.98	100
TC3-GO	98.59	99.99	100
Control	12.90	97.80	36.50

**Table 6:** Reduction of the microbiological load with *Staphylococcus aureus* ATCC 6538 (1.00 × 10^5^ CFU/mL) of unirradiated leathers, after irradiation and 60 days afterwards, TC and TC-GO samples, compared to control sample.

Sample	Leather Finished with Nanocomposites and Unirradiated	Leather Finished with Nanocomposites and Irradiated	Leather Finished with Nanocomposites and Irradiated, After 60 Days
	Reduction, %	Reduction, %	Reduction, %
TC1	96.92	99.82	99.91
TC2	93.90	99.92	100
TC3	94.92	99.95	100
TC1-GO	94.92	99.97	100
TC2-GO	94.92	99.85	99.99
TC3-GO	97.88	99.96	100
Control	17.65	97.66	40.00

## Data Availability

The original contributions presented in this study are included in the article/Supplementary Materials. Further inquiries can be directed to the corresponding authors.

## Supplementary Materials

The following supporting information can be downloaded at: https://www.mdpi.com/article/10.3390/ma19112358/s1, SI: Table S1. Framework technology for leather coating with nanocomposites; Figure S1. Leather surfaces coated with: TA nanocomposites before (a) and after irradiation (b); TA-GO nanocomposites before (c) and after irradiation (d); TC nanocomposites before (e) and after irradiation (f); TC-GO nanocomposites before (g) and after irradiation (h); Table S2. Microbiological load with *Escherichia coli ATCC 25922* (1.00 × 10^5^ CFU/mL) of unirradiated leathers, after irradiation and 60 days after irradiation, TA and TA-GO ranges; Table S3. Microbiological load with *Staphylococcus aureus ATCC 6538* (1.00 × 10^5^ CFU/mL) of unirradiated leathers, after irradiation and 60 days after irradiation, TA and TA-GO ranges; Table S4. Microbiological load with *Escherichia coli ATCC 25922* (1.00 × 10^5^ CFU/mL) of unirradiated leathers, after irradiation and 60 days after irradiation, TC and TC-GO ranges; Table S5. Microbiological load with *Staphylococcus aureus ATCC 6538* (1.00 × 10^5^ CFU/mL) of unirradiated leathers, after irradiation and 60 days after irradiation, TC and TC-GO ranges; Figure S2. SEM images (200×, 60,000×, 120,000×) of surface morphology with elemental composition (EDS) and particle sizes distribution of nanocomposites on leather surfaces: TA2, before (a, a’) and after gamma irradiation (b, b’); TA3, before (c) and after gamma irradiation (c’); TA3-GO, before (d) and after gamma irradiation (d’); TC2, before (e) and after gamma irradiation (e’); TC3, before (f, f’) and after gamma irradiation (g, g’); TC2-GO, before (h) and after gamma irradiation (h’); Figure S3. Color differences of MB stain exposed to Vis light and measured for irradiated and unirradiated leather surfaces coated with (a) TA1, (b) TA1-GO, (c) TA2, (d) TA2-GO, (e) TC1, (f) TC1-GO, (g) TC2 and (h) TC2-GO nanocomposites.
