# Supplementary material for: Enhanced Activity of Leather Materials Coated with Silver, Copper and Graphene Oxides-Decorated TiO2 Nanocomposites and Gamma Irradiated
Source: Materials (Basel). 2026 Jun 2;19(11):2358. doi: 10.3390/ma19112358 (PMC13258489; doi:10.3390/ma19112358)
Supplement: Supplementary file 1 [file materials-19-02358-s001.zip › materials-4059020-supplementary.pdf]

Supplementary information

Enhanced Activity of Leather Materials Coated with Silver, Copper and Graphene Oxides-Decorated TiO2 Nanocomposites and Gamma Irradiated

Carmen Gaidau [1,*], Cosmin Alexe [1], Rodica Roxana Constantinescu [1], Laurentiu Dinca [1], Ioana Stanculescu [2,3,*], Mihalis Cutrubinis [3] and Dragoș Cosma [4]

[1] The Research and Development National Institute for Textiles and Leather, 16, Lucretiu Patrascanu Street, 030508 Bucharest, Romania; cosminandrei.alexe@yahoo.com (C.A.); rodica.roxana@yahoo.com (R.R.C.); laurentiu.dinca@incdtp.ro (L.D.)

[2] Department of Physical Chemistry, University of Bucharest, 4–12 Regina Elisabeta Bd., 030018 Bucharest, Romania

[3] Horia Hulubei National Institute of Research and Development for Physics and Nuclear Engineering, 30 Reactorului Str., 077125 Magurele, Romania; mcutrubinis@nipne.ro

[4] National Institute for Research and Development of Isotopic and Molecular Technologies, 67–103 Donat Street, 400335 Cluj-Napoca, Romania; dragos.cosma@itim-cj.ro

* Correspondence: carmen.gaidau@incdtp.ro (C.G.); istanculescu@nipne.ro (I.S.)

2.2. Leather Coating with Composite Nanoparticles and Gamma Radiation Treatment

Table S1. Framework technology for leather coating with nanocomposites

Sheepskin crust leathers	Nanocomposite layers and application			
Finishing materials	Layer, mL			Application
	1	2	3	
Compact acrylic base coat binder	25			Layer 1:
White pigment with nanocomposite (1%)	11			2 sprays, air drying,
Water	64			ironing at 50⁰C and at 100 atm
Compact acrylic base coat binder		25		Layer 2:
White pigment with nanocomposite (1%)		11		2 sprays with intermediary dryings
Water		64		
Nitrocellulose lacquer emulsion with nanocomposite (0.3%)			42	Layer 3:
				2 sprays with intermediary
Water			8	dryings
				Ironing at 50⁰C and 100 atm

3. Results and Discussions

3.1. Leather Coated with Nanoparticle Composites Based on TiO_2 Decorated with Ag, Cu_2O/CuO and Graphene Oxide

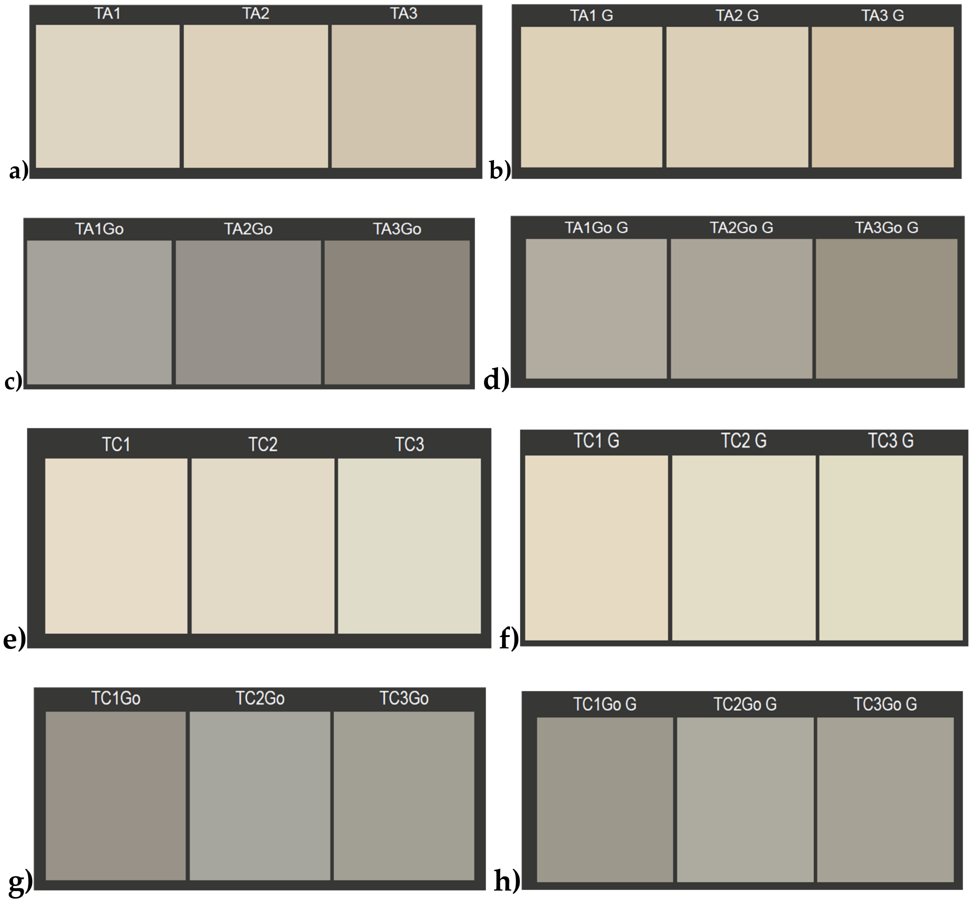

Figure S1. Leather surfaces coated with: TA nanocomposites before (a) and after irradiation (b); TA-GO nanocomposites before (c) and after irradiation(d); TC nanocomposites before (e) and after irradiation (f); TC-GO nanocomposites before (g) and after irradiation (h).

3.3. Antimicrobial Activity of Leather Surfaces before and After Gamma Radiation Treatment

Table S2. Microbiological load with *Escherichia coli ATCC 25922* (1.00 x 10^5 CFU/mL) of non-irradiated leathers, after irradiation and 60 days after irradiation, TA and TA-GO ranges. The values are the average of three sample measurements, with standard deviations and a statistical confidence level of $p < 0.05$.

Sample	Leather finished with nanocomposites and non-irradiated	Leather finished with nanocomposites and irradiated	Leather finished with nanocomposites and irradiated**, after 60 days**
	CFU/mL	CFU/mL	CFU/mL

TA1	$4.55x10^3$± 2.88	$1.72x10^2$± 2.65	$4.1x10^1$± 2.00	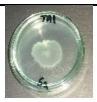
TA2	$5.30x10^3$± 1.00	9.1 $x10^1$± 2.08	1.00±0.57	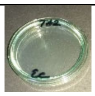
TA3	$2.24x10^3$± 3.05	5.5 $x10^1$± 0.57	1.00±0.57	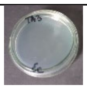
TA1-GO	$2.53x10^3$± 1.53	3.7 $x10^1$± 2.08	0	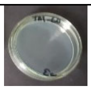
TA2-GO	$2.40x10^3$± 2.00	3.8 $x10^1$± 0.57	0	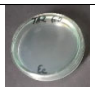
TA3-GO	$2.19x10^3$± 2.51	$5.4x10^1$± 1.53	0	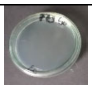
Control	$2.7x10^4$± 0.57	$7.4x10^4$± 2.65	$6.35x10^4$± 0.57	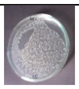

Table S3. Microbiological load with *Staphylococcus aureus ATCC 6538* (1.00 x 10^5 CFU/mL) of non-irradiated leathers, after irradiation and 60 days after irradiation, TA and TA-GO ranges. The values are the average of three sample measurements, with standard deviations and a statistical confidence level of $p < 0.05$.

Sample	Leather finished with nanocomposites and non-irradiated	Leather finished with nanocomposites and irradiated	Leather finished with nanocomposites and irradiated**, after 60 days**	
	CFU/mL	CFU/mL	CFU/mL	
TA1	$4.55x10^3$± 2.08	$1.85x10^2$± 1.53	$8.7x10^1$± 2.08	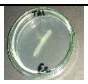
TA2	$5.30x10^3$± 0.88	8.00 $x10^1$± 2.31	0	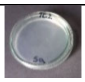
TA3	$2.24x10^3$± 2.51	5.30 $x10^1$± 1.00	0	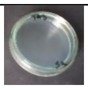
TA1-GO	$2.53x10^3$± 1.53	2.08 $x10^1$± 1.53	0	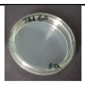
TA2-GO	$2.40x10^3$± 2.08	1.53 $x10^1$± 2.08	0	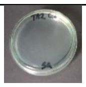
TA3-GO	$2.19x10^3$± 1.15	$4.30x10^1$± 2.08	0	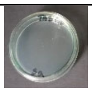

Control	$2.8x10^4$± 0.58	$2.304x10^3$± 0.58	6.00 $x10^4$±0.57	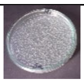

Table S4. Microbiological load with *Escherichia coli ATCC 25922*(1.00 x 10^5 CFU/mL) of non-irradiated leathers, after irradiation and 60 days after irradiation, TC and TC-GO ranges. The values are the average of three sample measurements, with standard deviations and a statistical confidence level of $p < 0.05$.

Sample	Leather finished with nanocomposites and non-irradiated	Leather finished with nanocomposites and irradiated	Leather finished with nanocomposites and irradiated**, after 60 days**	
	CFU/mL	CFU/mL	CFU/mL	
TC1	$1.57x10^3$± 4.95	$1.16x10^2$± 1.41	0.00	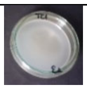
TC2	$8.05x10^2$± 1.00	5.90 $x10^1$± 2.31	0.00	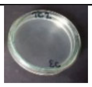
TC3	$9.70x10^2$± 1.15	2.10 $x10^1$± 351	0.00	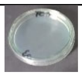
TC1-GO	$4.56x10^3$± 1.41	6.90 $x10^1$± 1.41	0.00	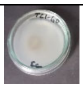
TC2-GO	$7.30x10^3$± 2.64	2.30 $x10^1$± 1.41	2.00±1.15	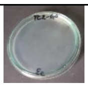
TC3-GO	$1.41x10^3$± 1.42	7.00± 1.15	3.00±2.8	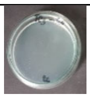
Control	$2.7x10^4$± 2.83	$2.20x10^3$± 1.41	$6.35x10^4$± 0.08	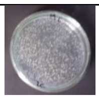

Table S5. Microbiological load with *Staphylococcus aureus ATCC 6538* (1.00 x 10^5 CFU/mL) of non-irradiated leathers, after irradiation and 60 days after irradiation, TC and TC-GO ranges. The values are the average of three sample measurements, with standard deviations and a statistical confidence level of $p < 0.05$.

Sample	Leather finished with nanocomposites and non-irradiated	Leather finished with nanocomposites and irradiated	Leather finished with nanocomposites and irradiated**, after 60 days**	
	CFU/mL	CFU/mL	CFU/mL	
TC1	$3.08x10^3$±4.95	$1.85x10^2$±1.41	2.00±0.71	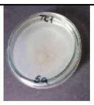

TC2	$6.10x10^3±1.00$	$8.00\ x10^1±2.31$	2.00±0.57	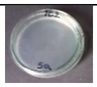
TC3	$6.74x10^3±1.15$	$5.30\ x10^1±3.51$	0.00	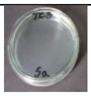
TC1-GO	$3.46x10^3±1.41$	$2.08\ x10^1±1.41$	0.00	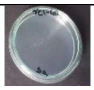
TC2-GO	$5.08x10^3±2.64$	$1.53\ x10^1±1.41$	14.00±1.15	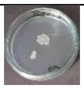
TC3-GO	$2.41x10^3±1.41$	$4.30x10^1±1.15$	0.00	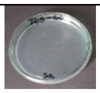
Control	$2.80x10^4±2.83$	$2.34x10^3±1.41$	$6.00x10^4±0.71$	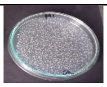

3.4. Morphology and Composition Analyses by SEM/EDS

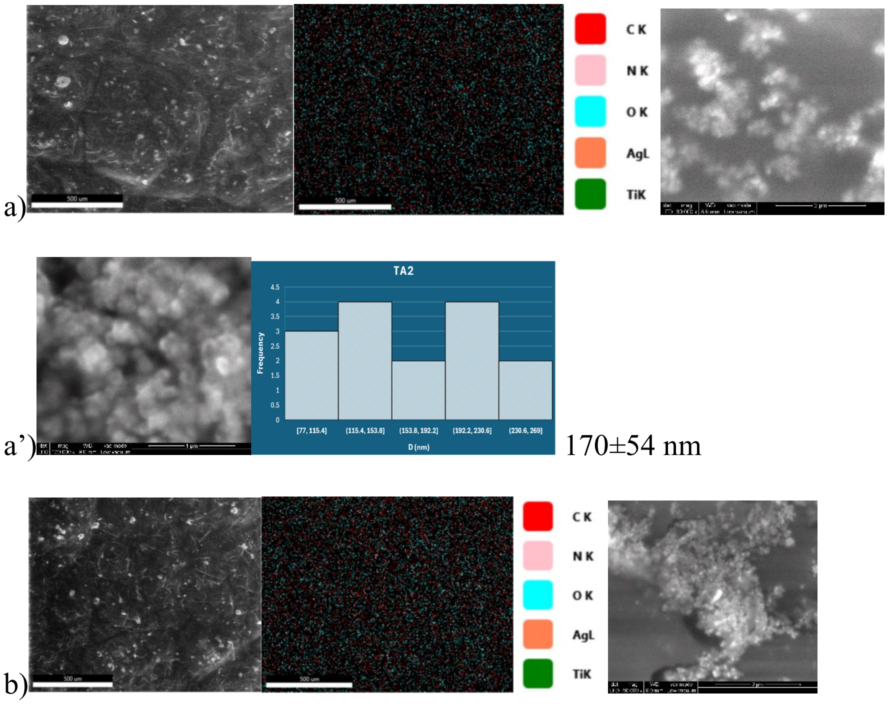

b')
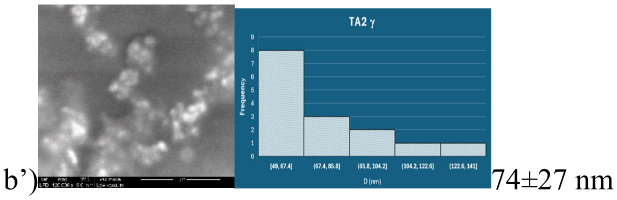

74±27 nm

c)
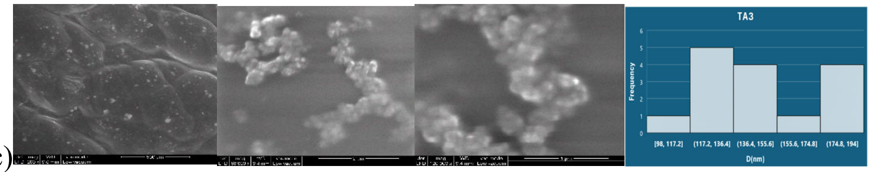

150±28 nm

c')
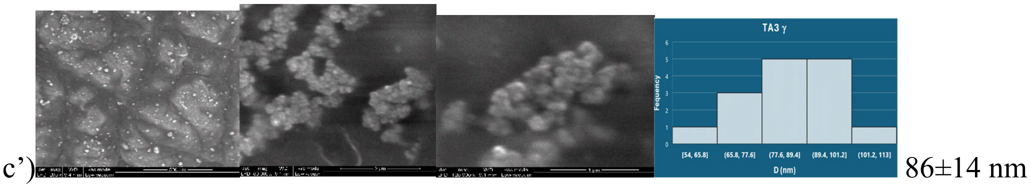

86±14 nm

d)
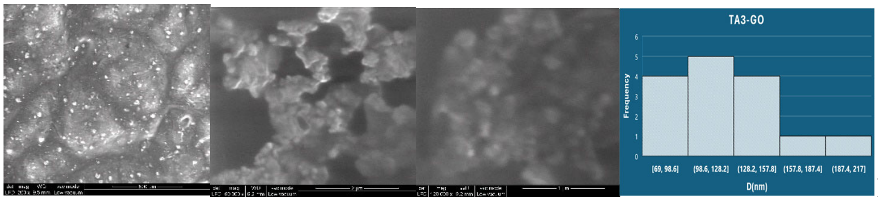

126±35 nm

d')
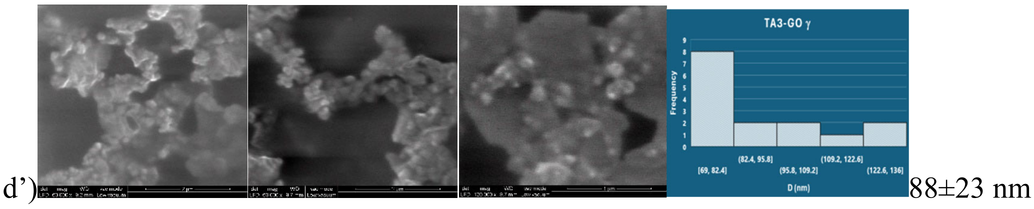

88±23 nm

e)
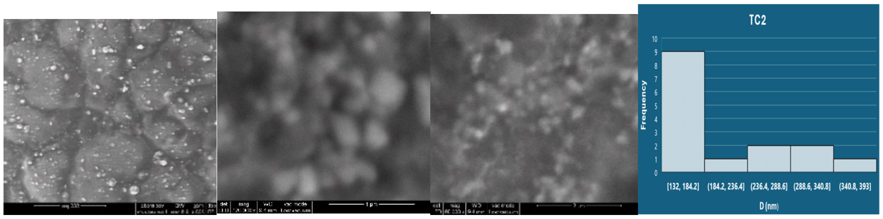

212±75 nm

e')
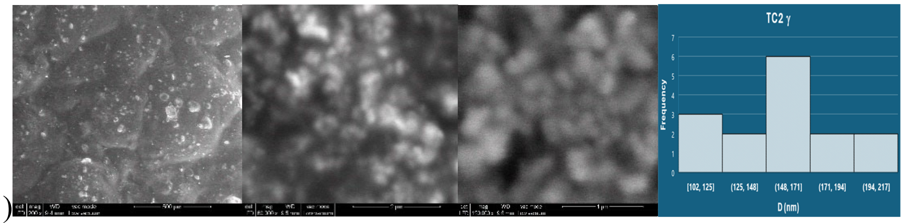

154±33 nm

f)
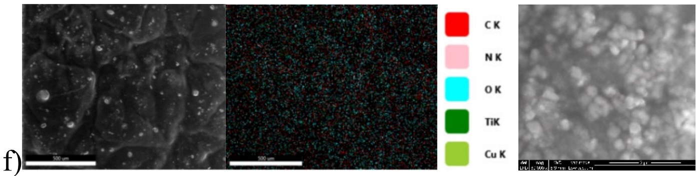

f')
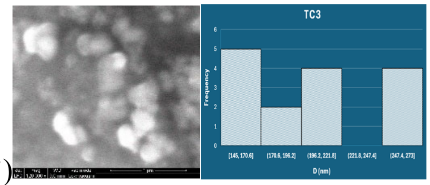

203±45 nm

g)
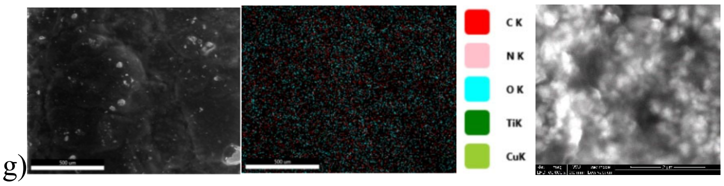

g')
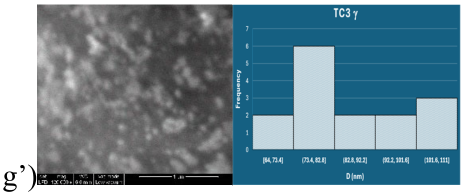

87±15 nm

h)
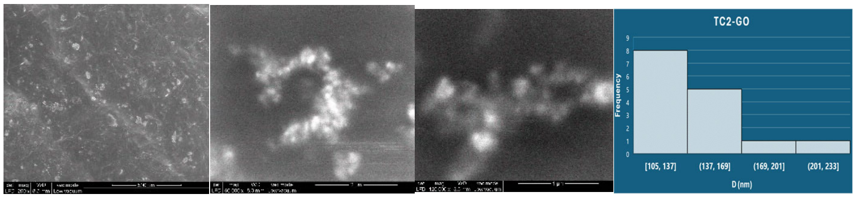

139±32 nm

h')
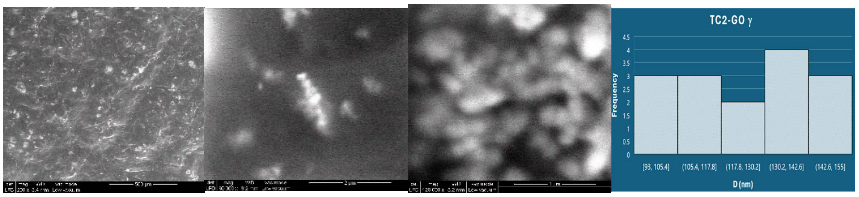

123±20 nm

Figure S2. SEM images (200x, 60,000c, 120,000x) of surface morphology with elemental composition (EDS) and particle sizes distribution of nanocomposites on leather surfaces: TA2, before (a, a′) and after γ irradiation (b, b′); TA3, before (c) and after γ irradiation (c′);TA3-GO, before (d) and after γ irradiation (d′), TC2, before (e) and after γ irradiation (e′), TC3, before (f, f′) and after γ irradiation (g,g′); TC2-GO, before (h) and after γ irradiation (h′).

3.5. Photocatalytic decomposition of organic stains under visible light

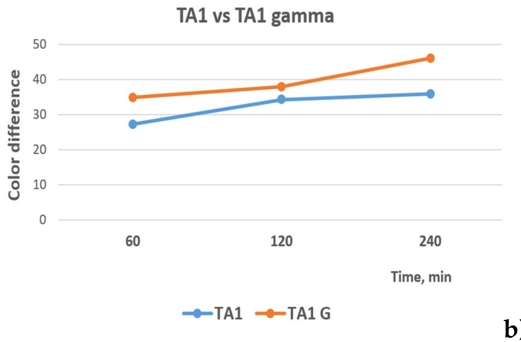

a)

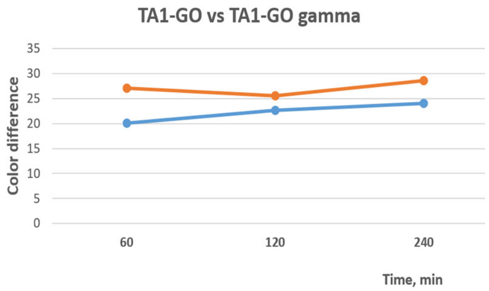

b)

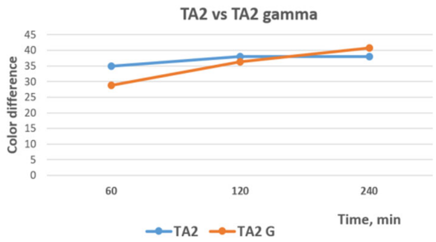

c)

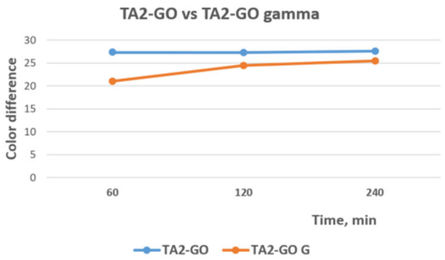

d)

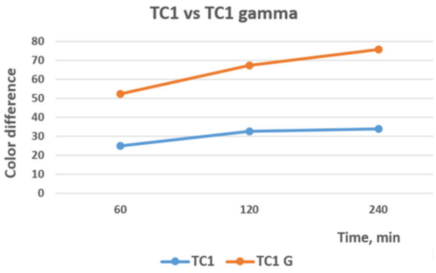

e)

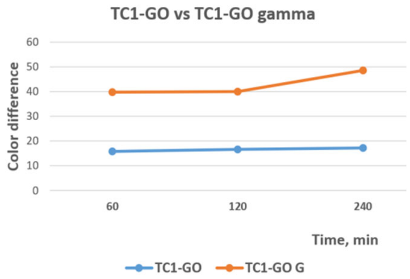

f)

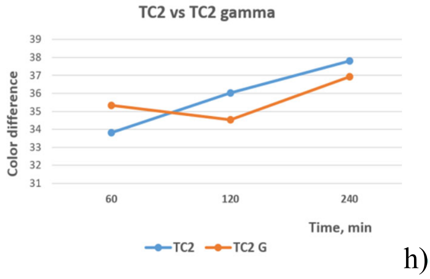

g)

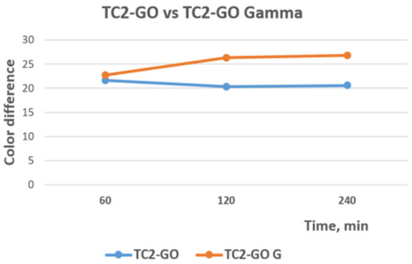

h)

Figure S3. Color differences of MB stain exposed to vis light and measured for irradiated and non-irradiated leather surfacescoated with a) TA1, b) TA1-GO, c)TA2, d) TA2-GO, e) TC1, f) TC1-GO, g) TC2 and h)TC2-GO nanocomposites. (G- Gamma)
